# FTO1/362: Using Neural Nets in Medical Decision Making

**DOI:** 10.2196/jmir.1.suppl1.e26

**Published:** 1999-09-19

**Authors:** B Kanagaratnam, S Lavelle, R Comerford

**Affiliations:** ^1^National University of IrelandGalwayIreland

**Keywords:** Expert System, Neural Net, Computer-Aided Diagnosis

## Abstract

**Introduction:**

Providing medical doctors with an expert system in diagnosing diseases will help, especially the Junior doctors, in the enhancement of their decision making skills. The objective of this project was to test the diagnostic accuracy of a Neuroshell model for Gallstones disease and Ductal cancer.

**Methods:**

314 Jaundice related cases were collected on forms from staff in the Galway hospital. Using the FoxPro application, a database was created to store these cases. The database records included 6 diseases and 92 symptoms. The symptoms were entered as Y, N, or S. (Y = yes, N = no, and S = unknown). From the main database we have selected equal number of cases for the two diseases of interest: Gallstones and Ductal Cancer. A total of 136 cases were selected to form the Jaundice database. These cases were divided into a Train set (68 cases) and a Test set (68 cases). Conflicting cases were eliminated to improve the diagnostic accuracy. NeuroShell was instructed to learn the Train set and then was instructed to classify the Test set having the same or similar defining characteristics as the Train set. Codes for the Binary model were defined as follows: Y was converted to 1, N was converted to 0 and S was treated as 1, or 0. The latter definition gave poor diagnostic accuracy. Codes for the Analog model are as follows: Y was converted to 1, N was converted to 0 and S was converted to 0.5.

The model had 92 input fields and 2 output fields, and was trained using the train set for Gallstones and Ductal cancer. Then the model was tested with 68 cases that were not included in the training.

**Results:**

The diagnostic accuracy for the Train/Test sets is shown in Table 1 and Table 2 for the binary and analog models, respectively.

**Discussion:**

The NeuroShell model provides us with the ability to determine the probability and rating of a new case. The Analog model for the Test set showed a diagnostic accuracy of 79.5%. More cases are needed to make the the diagnostic system more effective. The NeuroShell model can provide the doctor with valuable information and assist her/him in optimising the approach to patient diagnosis and management.

**Table 1:** Binary Model Diagnostic Accuracy

	**Train Set**	**Test Set**
Gallstones	100 %	92%
Ductal Cancer	100 %	59%
Total Binary Model Accuracy	100 %	67%

**Table 2:** Analog Model of Diagnostic Accuracy

	**Train Set**	**Test Set**
Gallstones	100 %	89%
Ductal Cancer	100 %	70%
Total Analog Model Accuracy	100 %	79.5%

